# Mouse IgG2c Fc loop residues promote greater receptor-binding affinity than mouse IgG2b or human IgG1

**DOI:** 10.1371/journal.pone.0192123

**Published:** 2018-02-06

**Authors:** Daniel J. Falconer, Adam W. Barb

**Affiliations:** Roy J. Carver Department of Biochemistry, Biophysics and Molecular Biology Iowa State University, Ames, IA, United States of America; Russian Academy of Medical Sciences, RUSSIAN FEDERATION

## Abstract

The structures of non-human antibodies are largely unstudied despite the potential for the identification of alternative structural motifs and physical properties that will benefit a basic understanding of protein and immune system evolution as well as highlight unexplored motifs to improve therapeutic monoclonal antibody. Here we probe the structure and receptor-binding properties of the mouse IgG2c crystallizable fragment (Fc) to compare to mouse IgG2b and human IgG1 Fcs. Models of mIgG2c Fc determined by x-ray crystallography with a complex-type biantennary (to 2.05 Å) or a truncated (1)GlcNAc asparagine-linked (N)-glycan attached (to 2.04 Å) show differences in key regions related to mouse Fc γ receptor IV (mFcγRIV) binding. Mouse IgG2c forms different non-bonded interactions between the BC, DE and FG loops than the highly-conserved mIgG2b and binds to FcγRIV with 4.7-fold greater affinity in the complex-type glycoform. Secondary structural elements surrounding the Asn297 site of glycosylation form longer beta strands in the truncated mIgG2c Fc glycoform when compared to mIgG2c with the complex-type N-glycan. Solution NMR spectroscopy of the N-linked (1)GlcNAc residues show differences between mIgG2b, 2c and hIgG1 Fc that correlate to receptor binding affinity. Mutations targeting differences in mIgG2 DE and FG loops decreased affinity of mIgG2c for FcγRIV and increased affinity of mIgG2b. Changes in NMR spectra of the mutant Fc proteins mirrored these changes in affinity. Our studies identified structural and functional differences in highly conserved molecules that were not predicted from primary sequence comparison.

## Introduction

**Fig 1:**
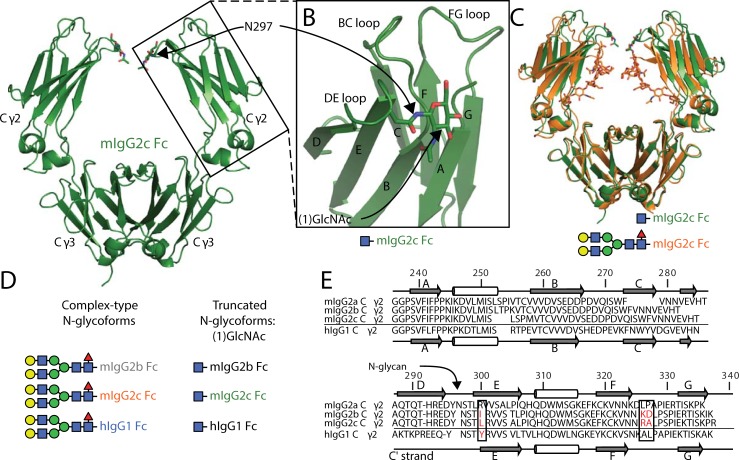
Mouse IgG2c Fc is comparable to other IgGs but shows differences in crucial features. **A**. A cartoon model of mouse IgG2c Fc solved by x-ray crystallography. Domain and secondary structure element labels (**B**.) are noted. **C**. An overlay of two mouse IgG2c Fc models with different N-glycan composition. **D**. A cartoon schematic showing the glycoforms studied here, individual carbohydrate residues are indicated by colored shapes according to the SNFG system [2]. The colors of individual Fcs will be used as indicated throughout the text to denote sequence and glycan variants. **E**. Sequence and secondary structure arrangement of the Fc Cγ2 domains.

Antibodies protect against invading pathogens and diseased tissue through a host of different mechanisms, including neutralization, antibody-dependent cell mediated cytotoxicity (ADCC), complement-mediated cytotoxicity, phagocytosis, and trogocytosis. ADCC, as the name implies, is triggered when an IgG-coated particle engages certain Fc γ receptor (FcγR)-expressing leukocytes. Phagocytosis and trogocytosis can likewise be triggered through FcγR-mediated mechanisms. FcγRs bind IgG through the invariant homodimeric crystallizable fragment (Fc) formed by the C-terminal halves of the gamma heavy chain (Fig 1). Receptor binding interactions are sensitive to the subclass of IgG, with human IgG1 and 3 eliciting a response at lower concentrations than IgG2 and 4 [1].

FcγRIIIa/CD16A is the primary receptor that triggers ADCC in humans. CD16A preferentially binds IgG1 and 3 over IgG2 and 4 [3], and is expressed as the only FcγR on natural killer cells, but is also expressed on some populations of monocytes/macrophages [4]. A related molecule, CD16B is expressed on neutrophils and potentiates similar responses. The murine adaptive immune is similar with IgG2 antibodies and mFcγRIV comparable to hIgG1 and hCD16A, respectively, though no hCD16B homolog is known in mouse [5]. Mouse FcγRIV is expressed on neutrophils and monocytes/macrophages and a recent study indicates mFcγRIV shares multiple unique functional features associated with hCD16A, reinforcing the identity of these two proteins as homologues [6]. Though numerous studies described the structure and function of human IgG1 and the IgG1 Fc:CD16A interaction (including but not limited to [7–20]), much less is known about the mIgG2 structure and the mIgG2:FcγRIV complex despite the central role for mice strains in developing and testing monoclonal antibody therapies. Different antibodies provide the potential to understand disparate functional solutions selected by evolution in antibodies that are highly conserved at the primary sequence level, despite the potential for subtle differences at the antibody/receptor interface between human and mouse, and are expected to inform future antibody development efforts.

Mice strains express three IgG2 subclasses: IgG2a, 2b and 2c that share 74–80% primary sequence identity in the hinge and Fc regions with greatest similarity in the receptor binding Cγ2 domains (Fig 1E). Mouse IgG2a is highly polymorphic [21] with less variability described for the heavy chains of mIgG2b and mIgG2c. While mouse IgG2b is encoded by a distinct gene and likely present in all mice, the Igh-1a and Igh-1b genes encoding the mIgG2a and 2c heavy chains, respectively, appeared to be allelic with dramatically different allele frequencies found in different strains [21]. Four structures of mIgG2a and 2b are available in the protein database, and no structures of mIgG2c have been described [22–25].

IgGs in all systems thus described require co-translational modification for effector function. A three amino acid sequon of Asn-(any non Pro residue)-Ser/Thr is modified by the oligosaccharyltransferase complex that adds a 14-residue carbohydrate to the sidechain nitrogen atom of Asn297 (an N-glycan). This carbohydrate is trimmed and extended by glycosylhydrolase and glycosyltransferase enzymes in the ER and the Golgi during protein secretion, resulting in the expression of antibodies that contain primarily a complex-type biantennary N-glycan that varies primarily in the amount of galactose at the two non-reducing termini [2]. In addition to the requirement of the N-glycan for FcγR interactions, the composition of the N-glycan also impacts receptor binding affinity, primarily CD16A [14, 19, 26]. The correlation between antibody N-glycan composition and receptor binding affinity can be explained by the work of our group that showed the N-glycan specifically stabilizes the hIgG1 Fc C'E loop and increasing the N-glycan length adds further stabilizing intramolecular contacts [16–18, 20, 27]. Thus, intramolecular contacts between N-glycan and polypeptide residues are critical features of antibody structure and receptor binding function. Probing differences in N-glycan structure and motion promises to provide insight into antibody structure/function relationships.

**Fig 2:**
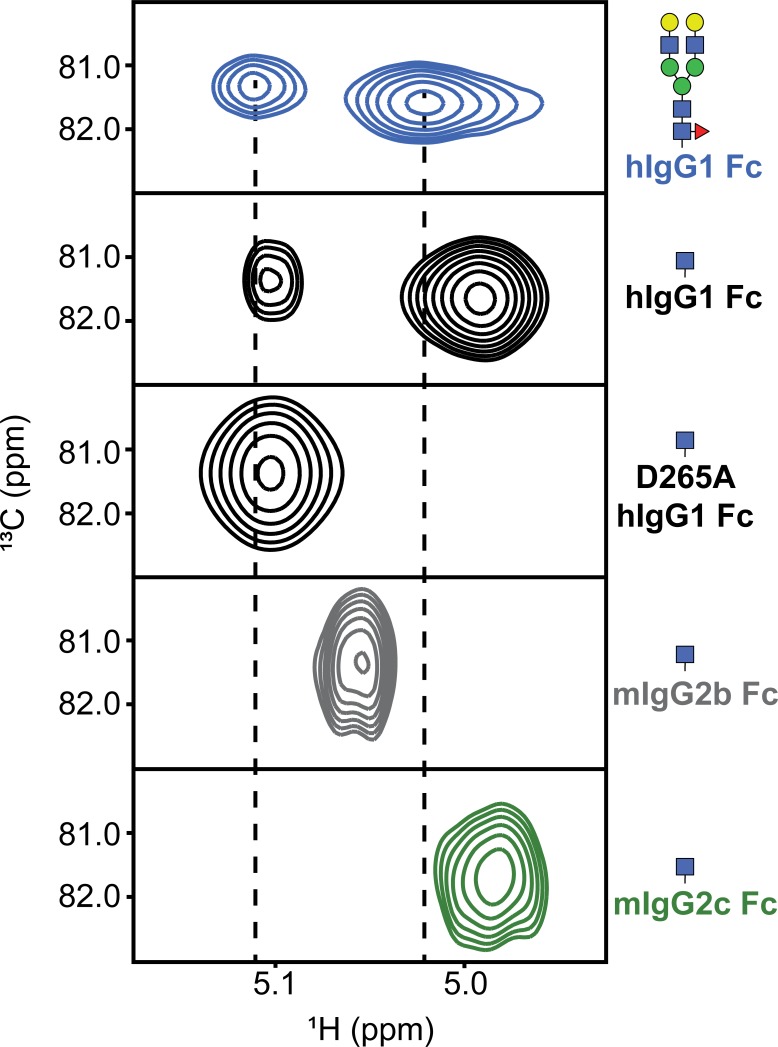
2d HSQC spectra of the Fc N-glycan (1)GlcNAc ^1^H1-^13^C1 reveals orthologous Fcs provide different N-glycan environments. The spectra of human IgG1 Fc were previously reported [20].

Our group recently reported a strategy to quickly identify interactions between N-glycans, in particular the asparagine-linked (1)GlcNAc residue, and polypeptide residues with 2d ^13^C-HSQC spectra of HEK293-expressed and ^13^C[glycan]-labeled glycoproteins [28]. We identified two CD16A N-glycans that contribute to antibody binding activity; the three remaining N-glycans showed little interaction. We also identified hIgG1 Fc residues that contribute to structural stabilization of proximal N-glycan residues and promote CD16A binding [20]. The ^1^H1-^13^C1 correlation on the hIgG1 Fc (1)GlcNAc residue showed two resolved peaks that were consistent with CD16A binding activity (Fig 2). Mutations that disrupted structure or glycosylation of the C'E loop caused the two peaks to collapse into one peak and likewise disrupted CD16A binding. N-glycan truncation to a single (1)GlcNAc residue preserved both the spectral features and receptor binding properties of hIgG1 Fc and simplified the NMR spectra. With this expression and NMR technique we investigated mouse antibody fragments and characterized differences in structure and function between mouse IgG2b and 2c Fc.

## Materials and methods

All materials purchased from Sigma-Aldrich unless noted.

### Protein expression and purification

Mouse Fc expression constructs were prepared by amplifying open reading frames corresponding to the Fc and hinge regions from heavy chain expression constructs for mIgG2b and 2c provided by Prof. Falk Nimmerjahn (University of Erlangen-Nuremberg). These fragments were cloned into the pGen2 vector *NotI* and *BamHI* sites [29] that encoded residues 224–447 for mIgG2b Fc, and mIgG2c Fc residues 224–447 or mIgG2c Fc residues 209-447(only used for crystallizing mIgG2c Fc with the truncated (1)GlcNAc N-glycan). Mouse Fc mutations were prepared with the fusion PCR protocol [30] and verified by DNA sequencing (ISU DNA sequencing facility). Mouse antibody fragments were expressed using HEK293F (Life Technologies) or HEK293S cells [31] grown in a mixture of 90% Freestyle293 (Life Technologies) and 10% ExCell media as previous described [29]. Fc expressions were purified on a 5 mL protein A-sepharose column eluted with 5x 5 mL 100 mM glycine, pH 3.0. Fractions were immediately neutralized with 2.5mL 1 M Tris, pH 8.0. Protein was exchanged into 20 mM MOPS, 100 mM sodium chloride, pH 7.2, using an Amicon 10 kDa cut off centrifugal filter and stored at 4°C.

DNA encoding mouse FcγRIV (residues Q19–D191) was cloned into the pGen2 vector using the *EcoRI* and *HindIII* sites to express an 8xHis and N-term GFP-tagged protein. Mouse FcγRIV was expressed in HEK293F cells grown in a mixture of 90% Freestyle293 (Life Technologies) and 10% ExCell media as described above and purified using a Ni-NTA agarose column (Qiagen). Purified receptor was exchanged into 20 mM MOPS 100 mM sodium chloride, pH 7.2, using an Amicon 10 kDa cut off centrifugal filter. FcγRIV was stored at -80°C after adding glycerol to a final (v/v) ratio of 25%.

### N-glycan remodeling

N-glycans from HEK293S(*lec*^*-/-*^)-expressed Fc were truncated using either endoglycosidase F1 or endoglycosidase S depending on the absence or presence of fucose on the Fc, respectively. Endoglycosidase was added in a 1:50 molar ratio to Fc in 20 mM MOPS, 100 mM sodium chloride, pH 7.2 for 18 h at RT. Digestion was assessed using SDS-PAGE(Supplementary Fig 3). Remodeled Fc (not 8xHis-tagged) was purified from endoglycosidase F1 (8xHis-tagged) by flowing over Ni-NTA agarose resin.

**Fig 3:**
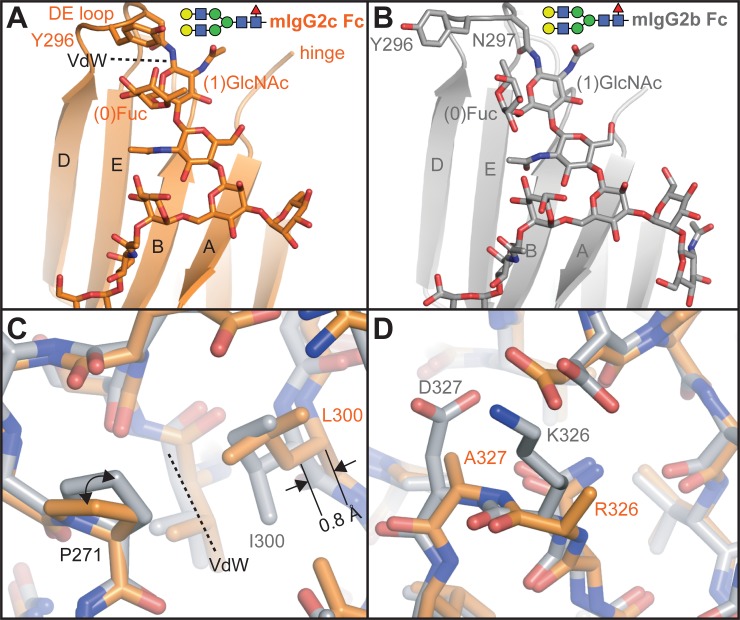
Mouse IgG2c Fc and 2b Fc show a high degree of similarity with a complex-type N-glycan, however, differences in loop residues impact conformation. **A**. Ribbon diagram of the mIgG2c Fc model shows clear Van der Waals contacts between Y296 and (0)Fuc. **B**. The ribbon diagram for mIgG2b Fc shows a different organization of the DE loop. **C**. A comparison of the 2b Fc and 2c Fc models shows the effect of residue 300 in DE loop conformation and **D**. sequence differences in the FG loop.

### Binding measurements

Mouse IgG2b Fc and IgG2c Fc were immobilized on a CM5 sensor chip using amine coupling on a Biacore T100 (GE Healthcare). Lane 1 was used for a control with no protein immobilized. Carboxymethyl dextran was activated by flowing over a 1:1 mixture of 0.4 M 1-ethyl-3(3-dimethylaminopropyl)carbodiimide and 0.1M N-hydroxy-succinimide for 7 min at 5 μL/min. Mouse IgG samples were diluted to a concentration of 1 μg/mL in 10 mM sodium acetate, pH 5.0, and flowed over the CM5 chip for 10 min at 5 μL/min. Unreacted sites were blocked with 1 M ethanolamine, pH 8.5, for 7 min. Typical immobilization response units varied from 400–1000. The CM5 chip used a coupling buffer containing 20 mM MOPS, 100 mM sodium chloride, and 0.05% P20 surfactant (GE Healthcare). The binding analysis buffer was identical but contained 1 μM bovine serum albumin. All experiments were carried out at 25°C. The CM5 chip was regenerated between runs with 100 mM glycine, pH 3.0, washes for 30 s to remove any bound receptor. Dissociation constants and rates were determined by averaging the values from at least two independent experiments. The error for this mean was determined by propagating the errors associated with the least-squares fit of each individual value. Differences in values were assessed by comparing the propagated error associated with each mean value.

### Crystallizing Mouse IgG2c Fc

Proteins were applied to a Superdex200 size exclusion column (GE Healthcare) pre-equilibrated with 20 mM MOPS, 100 mM sodium chloride, pH 7.2. Fractions containing Fc were identified using SDS-PAGE, pooled, and concentrated using a Amicon 10kDa cutoff centrifugal filter to 8–10 mg/mL. Initial screens for crystals were carried out at 18°C using the hanging drop vapor diffusion method. The mIgG2c Fc with a single (1)GlcNAc residue crystallized as large rod crystals when using the longer construct (residues 209–447) in 50 mM TRIS, pH 8.0, with 10% PEG 6K. Mouse IgG2c Fc with the complex-type glycan crystallized using the shorter construct (residues 224–447) as large rod crystals in 50 mM HEPES, 5% PEG 3.35K, pH 7.0.

### X-ray data collection and processing

Crystals were cryoprotected utilizing a quick soak in 20% glycerol (mIgG2c Fc with a truncated N-glycan) or 20% ethylene glycol (mIgG2c Fc with a complex-type N-glycan). Diffraction data was collected at the Advanced Photon Source at Argonne National Laboratory on beamline 23-IDD using a PILATUS detector. Data was indexed, merged and scaled using HKL-2000. Iterative molecular replacement was performed using Phenix Phaser-MR and the mIgG2b protein model (PDB ID: 2rgs; [24]). Final refinement was performed using Phenix with automatically-generated non-crystallographic symmetry restraints and translation-libration-screw restraints.

### NMR

NMR spectra were obtained using a 700-MHz Bruker Avance II spectrometer equipped with a 5 mm cryogenically cooled probe and operated with TopSpin 3.2. Samples were exchanged into a buffer containing 20 mM potassium phosphate, 100 mM sodium chloride, 0.5 mM DSS (4,4-dimethyl-4-silapentane-1-sulfonic acid), pH 7.0, using an Amicon 10kDa cutoff centrifugal filter. All HSQC spectra were collected at 50°C. Spectra were processed using NMRPipe and visualized using NMRViewJ (One Moon Scientific). Proton and carbon frequencies were referenced to the internal DSS standard using the methyl proton resonance (0.07 ppm).

### Accession numbers

Coordinates and structure factors have been deposited in the Protein Data Bank with accession numbers 6BHY (truncated) and 6BHQ (complex-type).

## Results

### Behavior of mouse IgG2 Fc differs from human IgG1 Fc

Spectra of ^13^C[glycan]-labeled mIgG2b Fc and mIgG2c Fc, following endoglycosidase truncation, showed a single peak for the (1)GlcNAc anomeric ^1^H1-^13^C1 correlation indicating differences in the chemical environment surrounding N297 (Fig 2). The peak from mIgG2c Fc showed a similar resonance frequency to the intense peak observed with hIgG1 Fc at ~4.99 ppm (^1^H). This intense peak was absent in a spectrum of hIgG1 Fc D265A that also showed no binding to CD16A [20]. The anomeric ^1^H peak from mIgG2b Fc appeared 0.07 ppm from the IgG2c Fc peak position and was comparable to a previously observation [32]. These spectra indicate mouse IgG2c and 2b share a similar single anomeric peak feature and the potential for less local structural heterogeneity than human IgG1 Fc which has a well characterized dynamic C'E loop and C' strand [18, 20]. Furthermore, the resonance frequency of the mIgG2c peak is shifted to a lower ^1^H ppm value than mIgG2b. Based on hIgG1 Fc, the lower ^1^H ppm value for mIgG2c Fc is consistent with greater conformational restriction and the potential for tighter FcγRIV binding compared to mIgG2b Fc.

### Mouse IgG2b and 2c bind FcγRIV with high affinity

Mouse IgG2c Fc bound mFcγRIV with 1.5-fold greater affinity than mIgG2b Fc when both contained core-fucosylated complex-type N-glycans (Table 1; Fig A in S1 File). These mouse antibody fragments bound mFcγRIV with 30–40 fold greater affinity (12–18 nM, respectively) than hIgG1 Fc bound FcγRIIIa/CD16A (550 nM [20]).

**Table 1:** Binding of mIgG2c Fc and mIgG2b Fc to mFcγRIV measured with surface plasmon resonance.

	(1)GlcNAc N-glycan		Complex-type N-glycan	
	*K*_d_ ± err (nM)		*K*_d_ ± err (nM)	
2b-wt	2800	±110	18.0	±0.3
2b-I300L	1800	±80	15.0	±0.2
2b-I300L/K326R/D327A	1300	±970	8.0	±0.1
2c-wt	600	±30	12.0	±0.2
2c-L300I	1000	±50	9.0	±0.1
2c-L300I/R326K/A327D	1100	±70	9.0	±0.1

Truncating the Fc N-glycan to display a single (1)GlcNAc residue reduced the binding of mIgG2c Fc by 50-fold, however, mIgG2c Fc displayed 4.7-fold greater binding than the similarly truncated mIgG2b Fc which was reduced by 155-fold following glycan truncation (Fig B in S1 File). This result is comparable to truncating the hIgG1 Fc N-glycan which reduced binding by 11-fold [20]. Thus, an extended complex-type biantennary N-glycan enhanced binding for both human IgG1 and mouse IgG2b and 2c Fcs.

### Structure of mouse IgG2c Fc in two glycoforms

We pursued the structure of these antibody fragments to define how differences in the amino acid primary sequence affect structure. Attempts to crystallize mIgG2c in both the full length and truncated N-glycoforms provided diffracting crystals (Table 2). Mouse IgG2b Fc truncated to the (1)GlcNAc residue did not form crystals in our screens; a high-resolution model of mIgG2b Fc with a full length N-glycan was previously reported [24]. Models of both mIgG2c Fcs in a truncated (pdb id 6BHY) or full-length (pdb id 6BHQ) N-glycoform proved similar to mIgG2b and mIgG2a Fcs with a homodimeric assembly and comparable secondary and tertiary structure elements (Fig 1A) [23, 24, 33]. Maps revealed clear electron density for all portions of the core Cγ2 and Cγ3 domains with one exception: the Cγ2 domain from chain B of mIgG2c Fc with a full-length N-glycan crystallized in an asymmetric unit without contacts stabilizing the chain B Cγ2 domain and some residues of the BC, DE and FG loops were unresolved despite high resolution diffraction (2.05 Å).

**Table 2:** Summary of crystallographic data.

	mIgG2c Fc N-glycoform	
	(1)GlcNAc	Complex-type
PDB id	6BHY	6BHQ
Wavelength (Å)	1.000	1.000
Resolution range (Å)	44.61–2.04 (2.113–2.04)*	46.08–2.05 (2.123–2.05)
Space group	C 1 2 1	P 21 21 21
Unit cell (*a b c*; Å)	103.233 89.215 69.851	67.735 73.399 118.408
(α β γ;°)	90.0 132.2 90.0	90.0 90.0 90.0
Total reflections	110252 (9915)	179382 (14723)
Unique reflections	29905 (2977)	37350 (3533)
Multiplicity	3.7 (3.3)	4.8 (4.2)
Completeness (%)	100 (100)	99 (95)
Mean I/σ(I)	9.55 (1.75)	16.9 (1.9)
Wilson B-factor (Å^2^)	33.22	40.55
R-merge	0.093 (0.553)	0.047 (0.489)
R-meas	0.109 (0.655)	0.052 (0.553)
CC1/2	0.995 (0.756)	0.999 (0.810)
Reflections used for R-free	6.3%	9.9%
R-work	0.2106 (0.2798)	0.2369 (0.3303)
R-free	0.2538 (0.3213)	0.2582 (0.3244)
Number of non-hydrogen atoms	3479	3551
macromolecules	3282	3160
ligands	34	198
water	163	193
protein residues	416	415
RMS bonds (Å)	0.019	0.020
RMS angles (°)	1.87	2.09
Ramachandran favored (%)	98.0	98.0
Ramachandran allowed (%)	2.2	1.8
Ramachandran outliers (%)	0	0.0
MolProbity Clash score	1.52	8.68
B-factors (Å^2^)		
average	38.4	55.01
macromolecules	38.16	53.94
ligands	58.45	78.14
solvent	39.10	48.8

* values in parentheses refer to the highest resolution shell

Two differences between mIgG2c Fc and mIgG2b Fc emerged upon comparing both models solved with a full-length N-glycan: a difference in quaternary structure and a difference in the DE loop. Mouse IgG2c showed a closer approach of the Cγ2 domains and a more open Cγ2/Cγ3 angle (Table B in S1 File). Though the Cγ2 domains accessed different orientations, the overall structural similarity was high with a low rmsd for backbone atoms (0.4 Å), though some differences were evident. The DE loop of mIgG2c Fc N-glycan was shifted 2.0 Å relative to the same loop for mIgG2b Fc (measured from Y296 in an overlay of the Cγ2 domains; Fig 3). The N-glycans of these Fcs occupied nearly identical interfaces with polypeptide sidechains, thus restricting the differences between the position of N297 Cα residues to 0.8 Å in an overlay of the Cγ2 domains. This DE loop displacement was due to the presence of a Leu residue at position 300 in mIgG2c Fc (mIgG2b I300) that forms van der Waals interactions with Pro271 and shifts the Cα of residue 300 by 0.8 Å and R293 by 1.3 Å further away from the E strand relative to mIgG2b (Fig 3C). It is also interesting to note van der Waals interactions between the Y296 sidechain and the (0)Fuc residue in mIgG2c Fc were not observed for mIgG2b.

**Fig 4:**
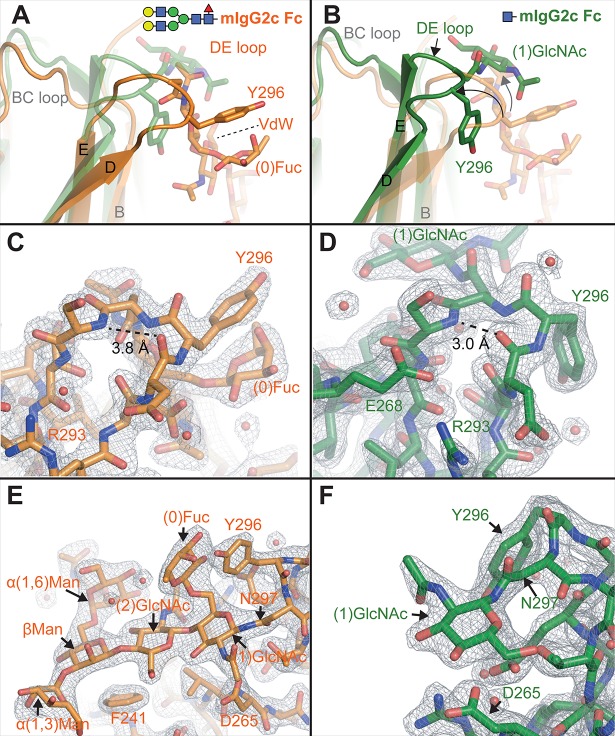
Truncating the mIgG2c Fc N-glycan alters the DE loop conformation. **A**-**B**) Comparison of mIgG2c Fc models with a complex-type N-glycan (orange ribbon) with a truncated (1)GlcNAc N-glycan (green ribbon). **C**-**D**) Electron density of the DE loops show significant displacements in the loop conformation (contoured to 1.5 σ). **E**-**F**) Electron density of the N-glycans show the usual contacts in the case of full length glycan, and a 180° rotation of the (1)GlcNAc residue in the truncated glycoform (contoured to 1.5 σ).

A model of the mIgG2c Fc truncated to a (1)GlcNAc N-glycan revealed substantial differences in the Cγ2 conformation and the DE loop (Fig 4). The Cγ2 domains of the mIgG2c Fc with the complex-type N-glycan and the truncated N-glycans compared with an rmsd of 0.5 Å. Beta character of the E strand extended by two residues which pulled the (1)GlcNAc residue of the truncated mIgG2c Fc upwards relative to mIgG2c with a full-length N-glycan, displacing the DE loop by 5.0 Å and shifting the BC loop by 1.5 Å for residues D269-P271 (Fig 4A). Crystal contacts were formed by DE-loop residues that would prevent the interconversion between the two distinct DE loop conformations observed in the crystals, however, based on the appearance of only one conformation from each glycoform despite screening crystals from multiple crystal conditions we believe the conformations presented here represent the predominant DE loop conformations sampled by each glycoform (Fig D in S1 File). Glycan truncation likewise impacted packing of the D strand against the E strand to form a beta sheet. This is evident from the carbonyl of D295 that is in hydrogen bond distance (3.0 Å) of the S298 amide. This distance increases to 3.8 Å and shifts the geometry away from a favorable hydrogen bond interaction in the structure containing the complex-type N-glycan. The truncated mIgG2c Fc glycoform also formed hydrogen bonds between E268, R293, and D295 further stabilizing the DE loop by coupling it to the BC loop.

This substantial reorganization was accompanied by a separation of the N-glycan from the stabilizing intramolecular interactions. The (1)GlcNAc residue and N297 sidechain rotated 180° relative the position found in mIgG2c with the full-length N-glycan. The low B-values (46 Å^2^) and resolution of this region are surprising (Fig 4D); the same (1)GlcNAc residue of hIgG1 Fc with the same truncation does not reorient, is less resolved and is characterized by higher B values (105 Å^2^) [34]. This comparison in conjunction with the NMR data above indicates that mIgG2c Fc contains a sequence motif that provides more interactions to stabilize this critical region than are present in hIgG1 Fc, however, the rearrangements upon truncation, including the extension of the E strand, likely do not promote receptor binding as mIgG2c is more sensitive to glycan truncation than human IgG1 Fc (50-fold and 11-fold reduction, respectively; Table 1).

### mIgG2b Fc mutations increase FcγRIV binding and affect N-glycan environment

Only three amino acid differences at residues 300, 326 and 327 distinguish the BC, DE and FG loop regions of mIgG2c Fc from mIgG2b Fc and these likely contribute to the mFcγRIV binding interface (Fig 1E). Indeed, the FcγRIV binding affinities are similar but mIgG2c binds with greater affinity than mIgG2b in either Fc glycoform studied here (Table 1). Residue I300 of mIgG2b Fc is positioned to perturb the DE loop organization and potentially reduce mIgG2b Fc affinity relative to mIgG2c. The two other differences at residues 326 and 327 do not appear to impact geometry of the FG loop backbone but may contribute to BC and DE loop motion by sidechain-mediated interactions (Fig 3D). The mIgG2b I300L mutation increased binding to FcγRIV by 1.2 to 1.6-fold for the truncated and complex-type glycoforms, respectively (Table 1). The inclusion of two FG loop mutations (mIgG2b I300L/K326R/D327A) increased binding from 2.2 to 2.3-fold.

**Fig 5:**
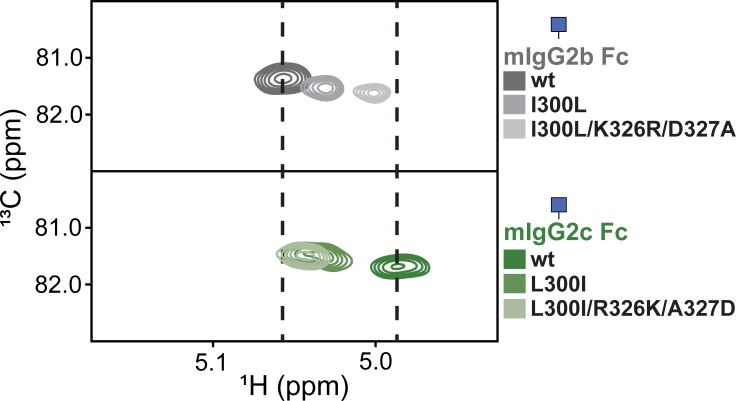
^1^H-^13^C HSQCs of anomeric (1)GlcNAc correlations from mouse IgG2b and mouse IgG2c Fc show near interconversion with amino acid substitution.

Changes in binding affinity mirrored NMR spectra of the mIgG2b Fc proteins, following N-glycan truncation (Fig 5). These spectra show the movement of anomeric peaks in an HSQC spectrum along a line towards the position of the same peak from the mIgG2c Fc protein. The reciprocal pattern of peak changes was observed with spectra of mIgG2c and the corresponding single L300I and triple L300I/R326K/A327D mutants, though the difference between single and triple mutants was less pronounced for the IgG2c mutations when compared to mIgG2b. The mutations decreased mIgG2c Fc binding by 1.8-fold for the truncated N-glycoform, and showed no difference for the complex-type N-glycoform outside of the error of the measurements (Table 1; on and off-rates for binding of the complex-type glycoforms are reported in Table A in S1 File).

It is worth noting that the FG loop mutations are at least 16 Å from the (1)GlcNAc residue, indicating changes to the FG loop affect the environment experienced by the N-glycan. Such interactions likely involve contacts between the loops that transfer structural information along a non-bonded network. These data demonstrate the partial interconversion of mIgG2b and mIgG2c structure and receptor binding affinity, and confirm the link between NMR spectra features, in particular the anomeric correlation spectra, and loop stabilizing interactions.

## Discussion

Here we demonstrate that mouse IgG2b and 2c have similar Fc structures for the complex-type N-glycoforms, however mIgG2c Fc binds FcγRIV with greater affinity. Differences in BC, DE and FG loop interactions account for the majority of the affinity difference. It is likely that other residues, not studied here including those at the Cγ2/Cγ3 interface and hinge, contribute to the difference in binding between the triple mutant protein IgG2b and wild-type IgG2c Fc. Surprisingly, mIgG2c formed a stable and well-resolved structure once the N-glycan was truncated to a single asparagine-linked (1)GlcNAc residue, unlike other Fcs investigated to date. Without a structure of the mIgG:FcγRIV complex it is impossible to know which conformation is more relevant for receptor binding, however it is likely the conformation observed with the tighter-binding mIgG2c Fc displaying a complex-type N-glycan is the more relevant conformation.

The effect of residue swaps on FcγRIV binding and the physical environment encompassing the anomeric (1)GlcNAc moiety highlights the link between DE loop stability and antibody function. Our group previously demonstrated that stabilizing the homologous loop in hIgG1 Fc on the μs-ms timescale increased receptor binding [18, 20]. Mouse IgG2c Fc encodes a structure that is more conducive to tighter receptor binding in both N-glycoforms studied here. Based on the structural features, similarity of NMR and binding analyses it is likely mIgG2c interactions restrict DE loop motion, preorganizing the Fc for receptor binding. Our data do not directly address the amplitude or timescales of motion present in the Fc DE loops of N-glycans.

**Fig 6:**
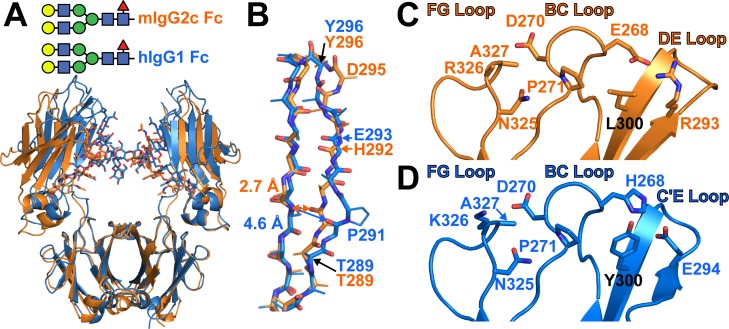
Mouse IgG2c and hIgG1 Fc show different strand and loop interactions. **A**. Mouse and human Fc fragments show a high degree of global similarity with an rmsd of 0.7 Å. **B**. P291 of hIgG1 disrupts a beta sheet formation in the C' strand, and shifts the register by one residue that is relieved by a bulge at mIgG2c D295. Differences and similarities are identified in residues that mediate loop interactions in mIgG2c Fc (**C**) and hIgG1 Fc (**D**).

A comparison of structures from orthologous antibodies has the potential to identify the range of structural solutions selected through evolutionary processes. In addition to the basic understanding of structure/function/evolution relationships, a complete understanding of these mechanisms will aid development of antibodies with superior therapeutic properties. It is curious that hIgG1 Fc binds CD16A with lower affinity than these mouse Fcs bind the orthologous mouse receptor FcγRIV. Though the overall organizations of the molecules are comparable and expected given the common origin, substantial differences in specific features emerged (Table B in S1 File). The C' strand of hIgG1 Fc (comparable to the D strand in mouse; Fig 1E) forms fewer stabilizing interactions with the E strand, resulting from distortion caused by a Pro residue at position 291 (Fig 6B). The distance between the 290 carbonyl oxygen and 303 amide nitrogen increased from 2.7 Å to 4.6 Å in human IgG1 Fc due to the Pro residue. The proline also shifts the register of the strand that is restored through a bulge at mIgG2c D295. Surprisingly, one reported model of mIgG2a forms a kink at position 291 similar to that of human IgG1 Fc [23] and another 2a model as well as 2b do not [24, 33]. The apparent structural plasticity of mIgG2a is surprising because the D strand sequence is identical to mIgG2c, suggesting other factors may contribute to structural differences in this area (Fig 1E).

More differences between hIgG1 Fc and mIgG2c Fc are found in the interactions between the BC, DE and FG loops. The impact of FG loop mutations on NMR spectra of the N-glycan clearly indicate a role for loop interactions in the mIgG2 Fc structures (Fig 5). Mouse IgG2c Fc shows a different organization around L300, including Van der Waals interactions with P271 and an ionic interaction between E268 and R293 (Fig 6C). The corresponding position in hIgG1 Fc (Y300) interacts with H268, P271, R292, R293 and E294 (Fig 6D). Human IgG1 Fc H268 is positioned to form an ionic interaction with E294 that is similar to the E268:R293 bridge in mIgG2c, though the protonation state of the H268 sidechain is not known. Numerous single mutations to the hIgG1 hinge, BC, C'E and FG loops are reported at positions 265, 267, 270, 297, 298 327, or 329 that reduced CD16A binding (reviewed in [35]). Some hIgG1 Fc combinations mutants bind CD16A with greater affinity than the wild type sequence and include variations in the C'E and FG loops, including G236A/S293A/A330L/I332E [36] and F243L/R292P/Y300L/V305I/P396L [37]. It is, however, unclear what the effect of each amino acid substitution, in particular Y300L found in mIgG2c, is on receptor binding.

It is unclear if the hIgG1 Fc C' strand destabilization is advantageous in the context of the human immune system, perhaps permanent stabilization through amino acid-amino acid contacts impedes the capacity for stabilizing intramolecular interactions between N-glycan and polypeptide residues to impact immune system function. For example, extending the hIgG1 Fc N-glycan increases intramolecular interactions between polypeptide and carbohydrate residues to reduce N-glycan motion, stabilize the C'E loop and increase receptor binding affinity [18, 20, 27]. There is ample evidence to implicate changes in hIgG1 N-glycan composition in multiple diseases and these data indicate composition may be actively monitored and modified by the body to tune immune system function [38–44]. Though Würher and coworkers recently described the mouse antibody N-glycome, comparable studies linking glycan composition with immune system response are not available as of this writing [45].

## Supporting information

S1 File Supporting information for the manuscript. (PDF)
