# Correction: Mouse IgG2c Fc loop residues promote greater receptor-binding affinity than mouse IgG2b or human IgG1

**DOI:** 10.1371/journal.pone.0196609

**Published:** 2018-04-24

**Authors:** The PLOS ONE Staff

The sequences appearing in the lower panel of Fig 1E are not properly aligned due to a technical error. Please see the complete, correct Fig 1 here. The publisher apologizes for the error.

**Fig 1:**
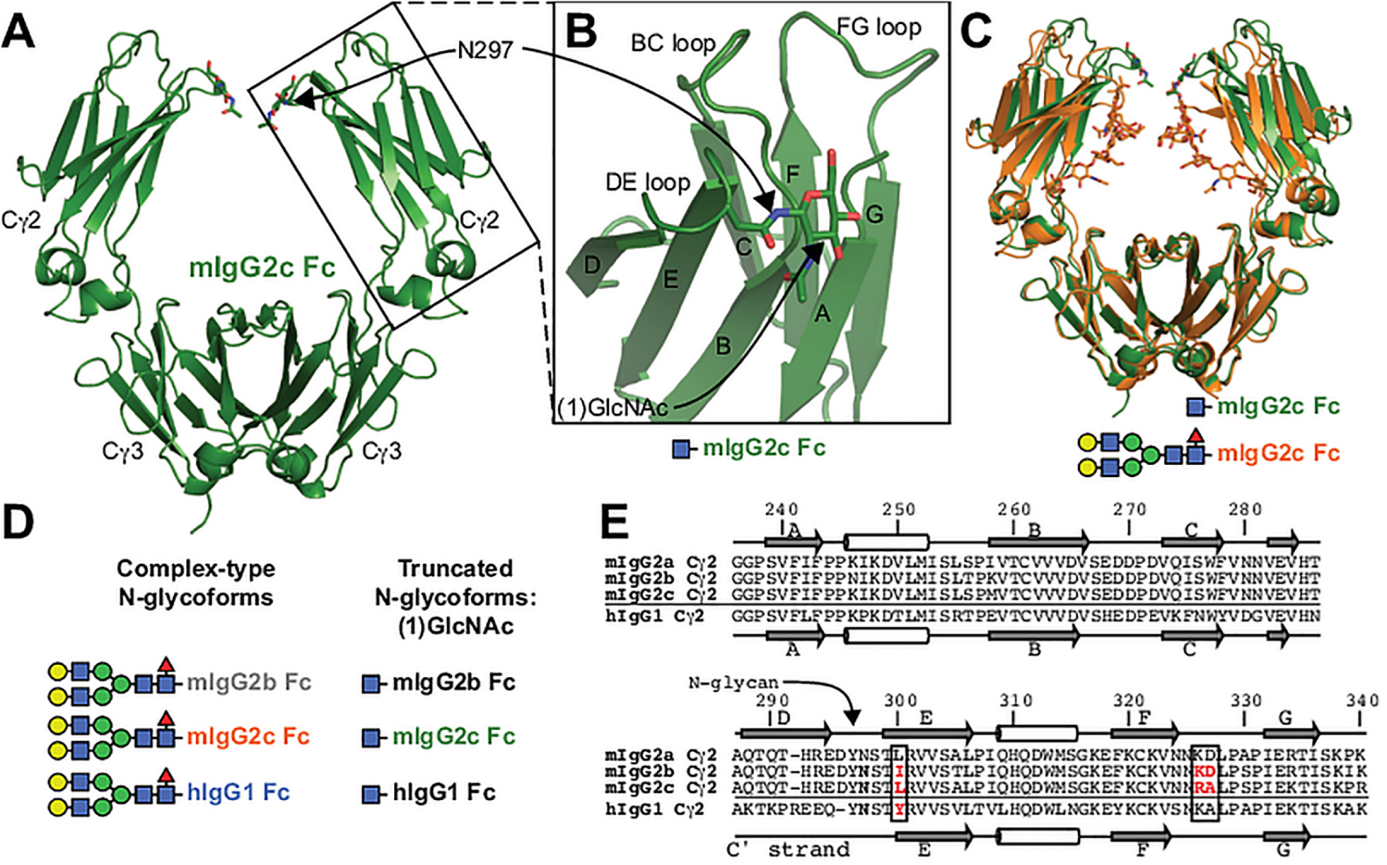
Mouse IgG2c Fc is comparable to other IgGs but shows differences in crucial features. **A**. A cartoon model of mouse IgG2c Fc solved by x-ray crystallography. Domain and secondary structure element labels (**B**.) are noted. **C**. An overlay of two mouse IgG2c Fc models with different N-glycan composition. **D**. A cartoon schematic showing the glycoforms studied here, individual carbohydrate residues are indicated by colored shapes according to the SNFG system [2]. The colors of individual Fcs will be used as indicated throughout the text to denote sequence and glycan variants. **E**. Sequence and secondary structure arrangement of the Fc Cγ2.
