# Supplementary material for: Mouse IgG2c Fc loop residues promote greater receptor-binding affinity than mouse IgG2b or human IgG1
Source: PLoS One. 2018 Feb 6;13(2):e0192123. doi: 10.1371/journal.pone.0192123 (PMC5800599; doi:10.1371/journal.pone.0192123)
Supplement: S1 File — (PDF) [file pone.0192123.s001.pdf]

Supplementary Info for:

Mouse IgG2c Fc loop residues promote greater receptor-binding affinity than mouse IgG2b or human IgG1

Daniel J. Falconer, and Adam W. Barb*

Roy J. Carver Department of Biochemistry, Biophysics and Molecular Biology

Iowa State University, Ames, IA, USA

* corresponding author email: abarb@iastate.edu

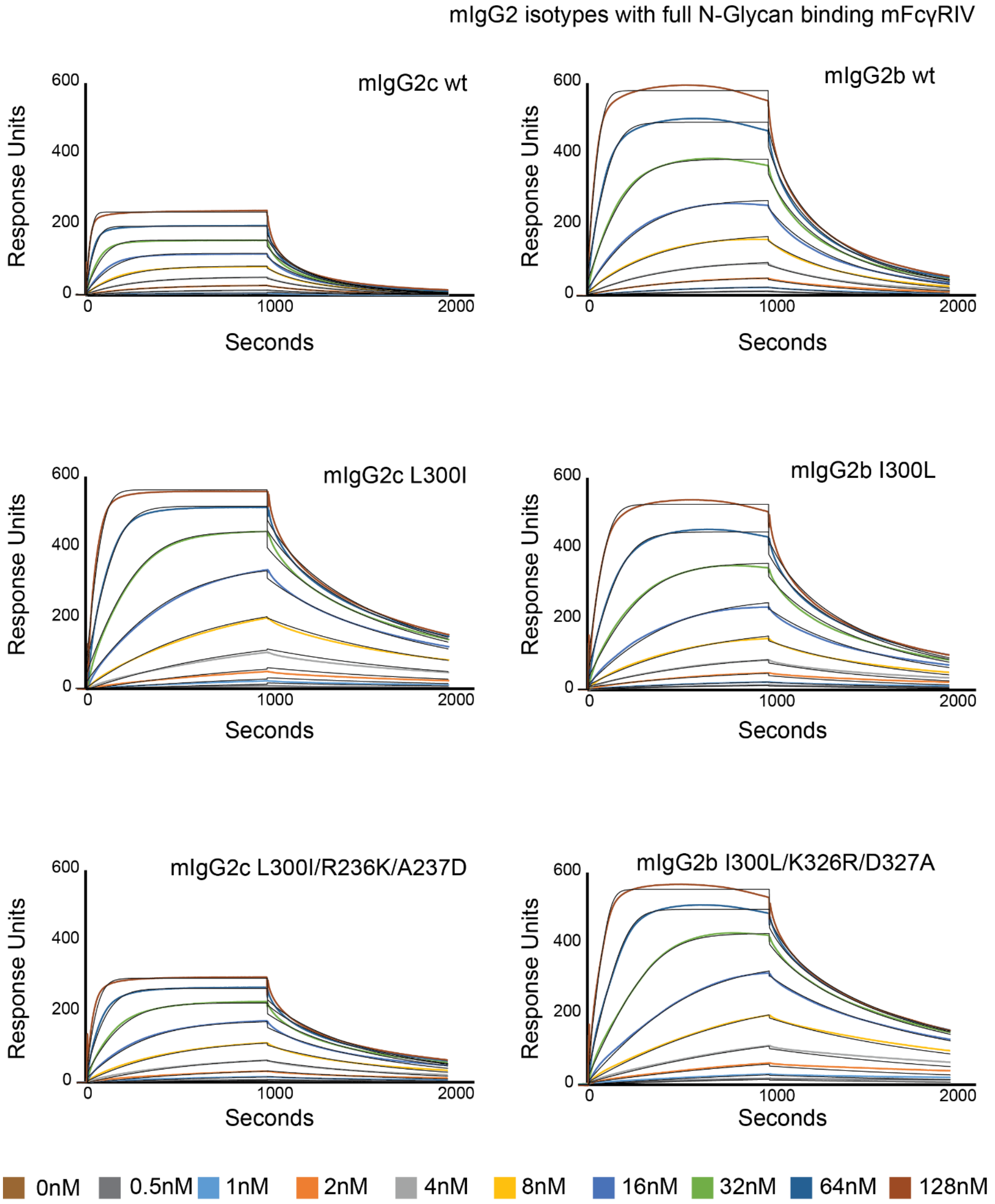

Fig A. SPR sensograms of mIgG2c isotypes with a full N-glycan at N297 binding with mFcγRIV. Kinetic models were fit to these SPR curves because clear equilibria were not established in all experiments. These data are representative of two independent experiments.

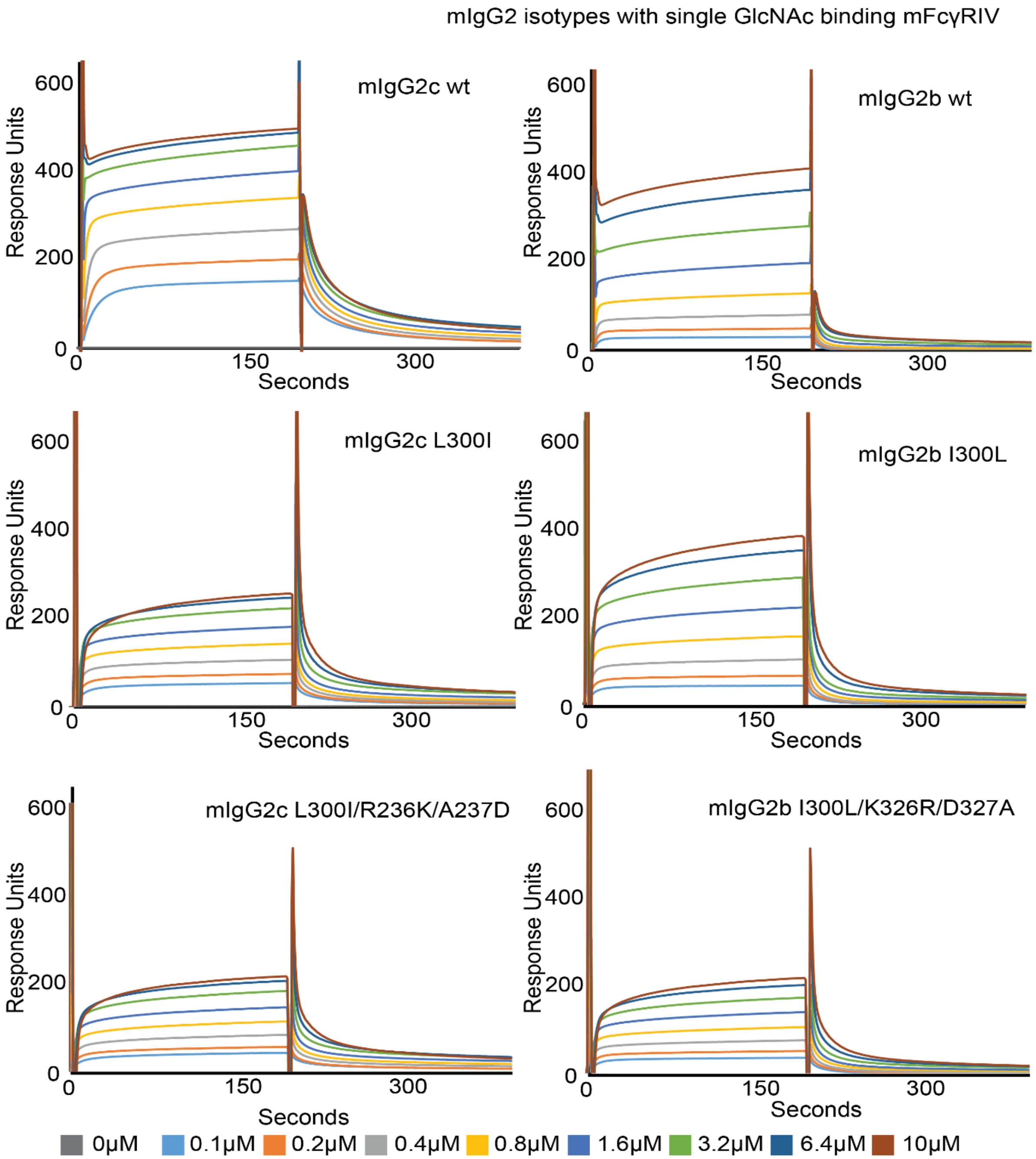

Fig B. SPR sensograms of mIgG2c isotypes with a full N-glycan at N297 binding with mFcγRIV. Equilibrium models were fit to these SPR curves. Error was calculated using least squares fitting. These data are representative of at least two independent experiments.

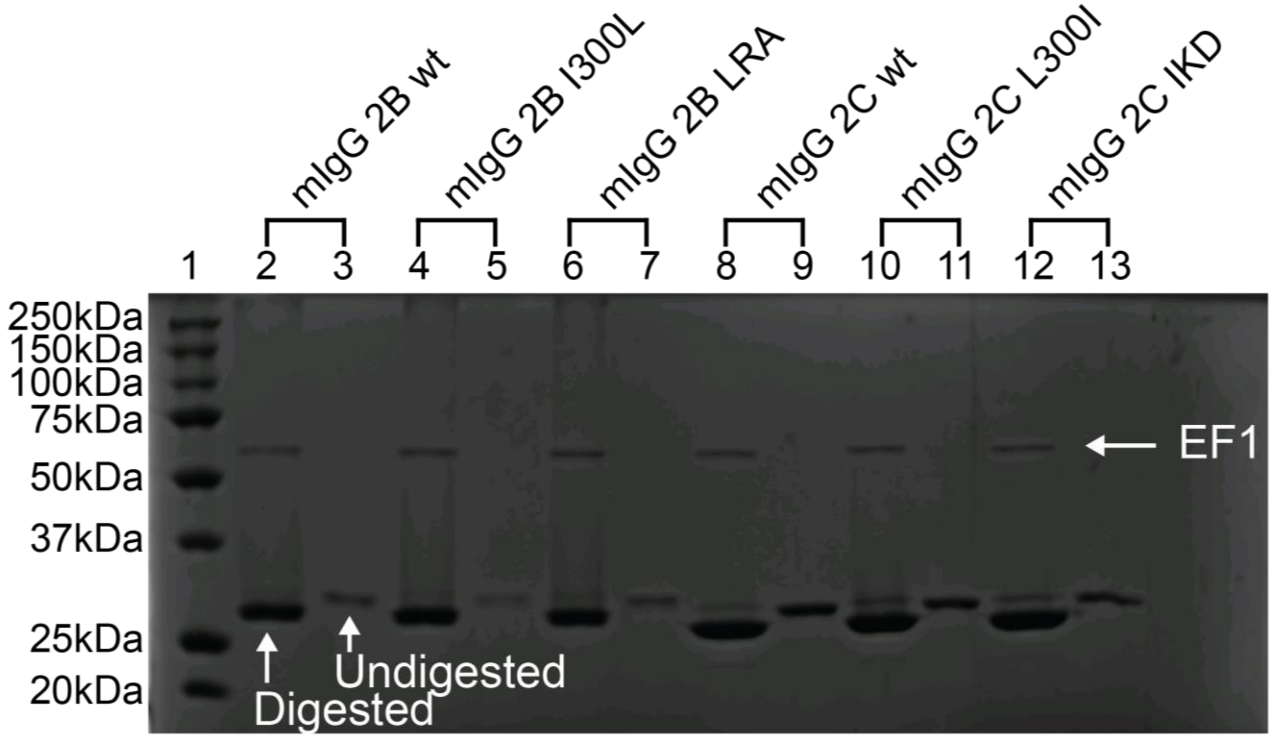

Fig C. Representative SDS-PAGE of endoglycosidase F1 (EF1) digestion of mIgG Fcs.

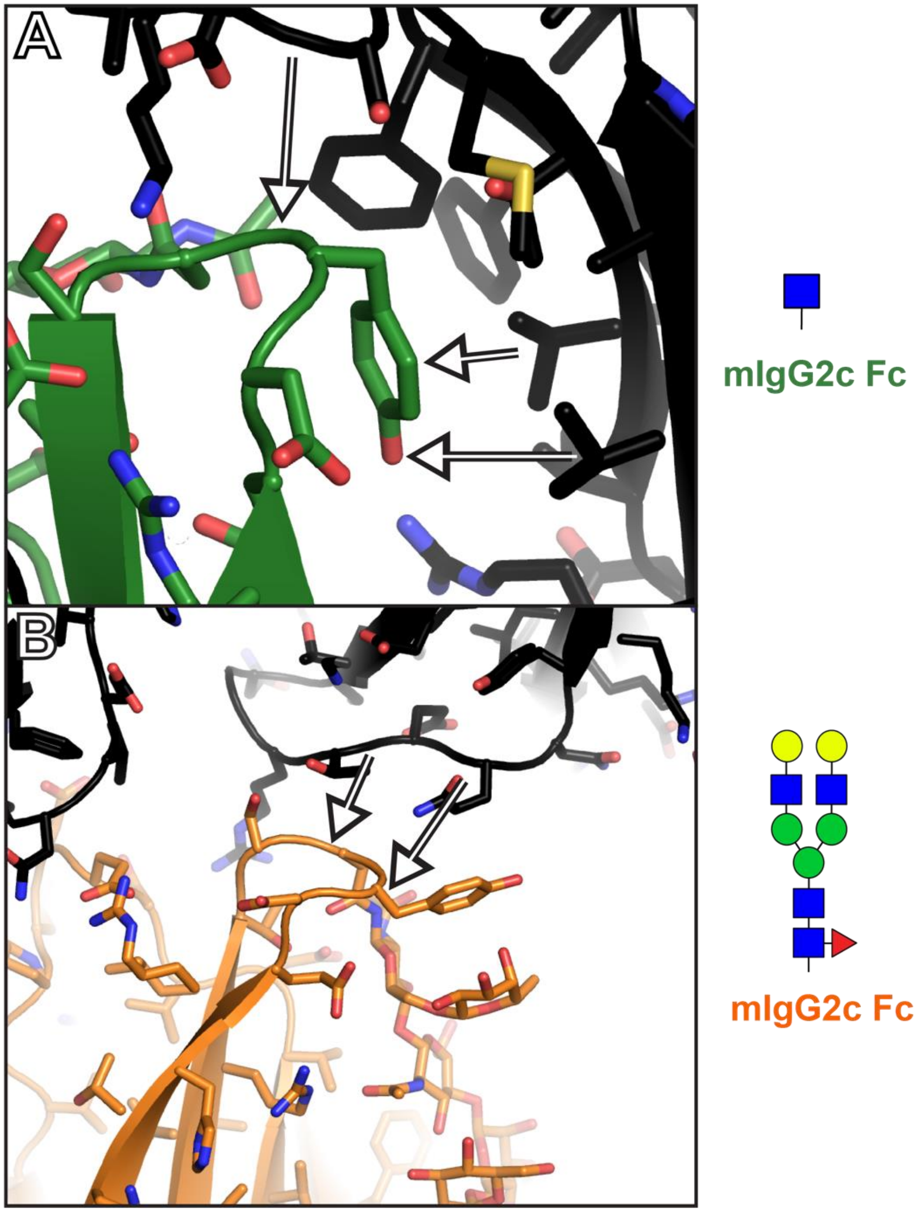

Fig D. Analysis of crystal contacts of the DE loop of mouse IgG2c. (A) DE loop contacts formed in the crystal of mIgG2c with the truncated N-glycan. Symmetry-related molecules are shown in *black*. (A) DE loop contacts formed in the crystal of mIgG2c with the full-length N-glycan.

Table A. Analysis of the kinetic parameters k_{on}, and k_{off} of the binding of mIgG2b and mIgG2c containing a complex-type glycan in complex with mFcγRIV. Data are representative of at least two independent experiments.

	k_{on} ($M^{-1}s^{-1}$)	error ($M^{-1}s^{-1}$)	k_{off} (s^{-1})	error (s^{-1})
2b-wt	4.0E+05	3.9E+03	7.4E-03	7.6E-05
2b-I300L	2.3E+05	1.7E+03	3.5E-03	2.7E-05
2b-LRA	4.2E+05	3.2E+03	3.5E-03	2.7E-05
2c-wt	6.8E+05	7.8E+03	8.3E-03	1.0E-04
2c-L300I	2.5E+05	1.4E+03	2.2E-03	1.2E-05
2c-IKD	2.3E+05	1.3E+03	2.1E-03	1.2E-05

Table B. Comparison of Fc quaternary structure according to the measurement points in (Frank J Mol Biol. 2014 Apr 17;426(8):1799-811. doi: 10.1016/j.jmb.2014.01.011). n.d.- not determined due to missing density.

PDB ID	Chain	Cγ2/Cγ3 angle	Cγ2/Cγ3 dihedral angle	N297/N297 distance (Å)
hIgG1 Fc	A	98.79	-31.02	29.5
pdb- 4ku1	B	95.25	-30.69	
mIgG2b Fc	A	83.72	-14.96	34.8
pdb- 2rgs	B	83.18	-15.18	
mIgG2c Fc	A	98.121	-11.51	27.1
(1)GlcNAc	B	95.6	-9.52	
mIgG2c Fc	A	92.88	-15.71	n.d.
complex-type	B	n.d.	n.d.	
